# Bilateral thalamic infarction with posterior cerebral artery variant (the arcade artery): A case report

**DOI:** 10.1097/MD.0000000000040991

**Published:** 2024-12-20

**Authors:** Langping Ling, Lingjia Xu, Yang Zhou

**Affiliations:** a Department of Emergency Internal Medicine, Shaoxing Second Hospital, Shaoxing, Zhejiang, China; b Department of Neurology, Shaoxing Second Hospital, Shaoxing, Zhejiang, China.

**Keywords:** case reports, infarction, paradoxical embolism, patent foramen ovale, posterior cerebral artery, thalamus

## Abstract

**Rationale:**

Bilateral thalamic infarction is a rare type of posterior circulation stroke, and it often presents with a reduced level of consciousness in the elderly. Arteriosclerosis is the primary etiology of bilateral thalamic infarction, including conditions such as native vessel stenosis or arterial-to-arterial embolism. Cardiogenic or paradoxical embolism can also lead to thrombosis of the perforator branches innervating the thalamus, and these emboli tend to disintegrate and lead to multiple lesions, even in elderly patients.

**Patients concerns:**

A 69-year-old man presented to our emergency room with sudden onset of drowsiness lasting for 2 days. A computed tomography scan revealed bilateral hypodense thalamic lesions, which resembled artery of Percheron infarcts. Magnetic resonance imaging confirmed cerebral infarction in the posterior circulation. Magnetic resonance angiography and diagnostic digital subtraction angiography suggested a suspicious embolus obstructing the junction between the arcade artery and the left posterior cerebral artery, which had further migrated. Echocardiography, 24-hour Holter monitoring, and deep venous ultrasonography were all negative; however, transesophageal echocardiography revealed a patent foramen ovale.

**Diagnosis:**

Paradoxical embolism is a rare occurrence in older adults. However, when considering the etiology of stroke in this patient, paradoxical embolism should remain a priority in the diagnostic process following a multifactorial risk assessment.

**Intervention:**

The patient was treated with antiplatelet, statin therapy, and foramen ovale closure.

**Outcome:**

He recovered well after the interventional closure surgery and is currently under follow-up.

**Lessons:**

The elderly experiencing a sudden drop in consciousness should be evaluated for thalamic lesions, primarily cerebral infarction in the posterior circulation. Anatomical artery variations may be helpful in attributing multiple and bilateral lesions to a single source of embolism. Digital subtraction angiography and transesophageal echocardiography can help to clarify the etiological categorization and formulate a secondary prevention strategy for cerebral infarction. Paradoxical embolism is a diagnostic dilemma in the elderly population, and treatment principles must be integrated with guidelines, the prospectively validated patent foramen ovale-associated stroke causal likelihood risk stratification system, interdisciplinary collaboration and customized analysis.

## 1. Introduction

The thalamus, a complex anatomical structure, its functional activity influences the limbic system, motor system, ascending reticular system, and cerebral cortex.^[1]^ Ischemic strokes in the posterior circulation that involve the thalamus can present with a variety of unusual and variable clinical manifestations.^[2,3]^ Therefore, it is crucial to have a thorough understanding of the thalamus’s anatomy and the vascular variants for accurate diagnosis and effective treatment,^[4]^ particularly when investigating the pathogenesis, as this is essential for developing a secondary prevention strategy for the patient.

Timely magnetic resonance imaging (MRI) can help analyze the degree of cerebral infarction in the posterior circulation, the involvement of vascular territories, and distinguish it from basilar artery occlusion. Cerebral angiography and transesophageal echocardiography are among the diagnostic protocols that can help clarify the etiological classification of posterior circulation infarction and inform the development of a secondary prevention plan for cerebral infarction.

This case involves a 69-year-old patient who was rushed to the emergency department due to sudden lethargy that lasted for 2 days. An emergency computed tomography (CT) scan revealed bilateral hypodense lesions in the thalamus, suggestive of a Percheron artery infarction. CT-angiography ruled out large vessel occlusion, while MRI confirmed a cerebral infarction in the posterior circulation, beyond the territory of the Percheron artery. Magnetic resonance angiography (MRA) and diagnostic digital subtraction angiography (DSA) showed suspected embolic occlusion at the junction between the arcade artery and the left posterior cerebral artery (PCA) with evidence of migration. Echocardiography, 24-hour Holter monitoring, and deep venous ultrasound all yielded negative results; however, transesophageal echocardiography suggested a patent foramen ovale (PFO) with high-risk characteristics.^[5]^ Clinically, it presents a diagnostic dilemma in determining the etiology of the stroke. The paradoxical embolism is considered a priority cause in this patient for several reasons. Firstly, the patient was performing a Valsalva maneuver at the time of the onset. Secondly, the examination revealed that the patient’s PFO morphology exhibited high-risk characteristics. Neuroimaging strongly suggested an embolic mechanism for the stroke, and the patient lacked other high-risk factors for stroke, such as significant atherosclerosis or atrial fibrillation. Although the decision to close the foramen ovale was supported by observational studies, it represented an attempt to obtain informed consent from the family beyond the existing guidelines and consensus.^[6,7]^ The patient has recovered well and is currently under follow-up.

It should be noted that paradoxical embolism is a rare cause of ischemic stroke in older adults. This case highlights the importance of screening older patients for PFO and investigating for thrombophilia, particularly when there are no other risk factors for stroke present.

## 2. Case report

**Figure 1.**
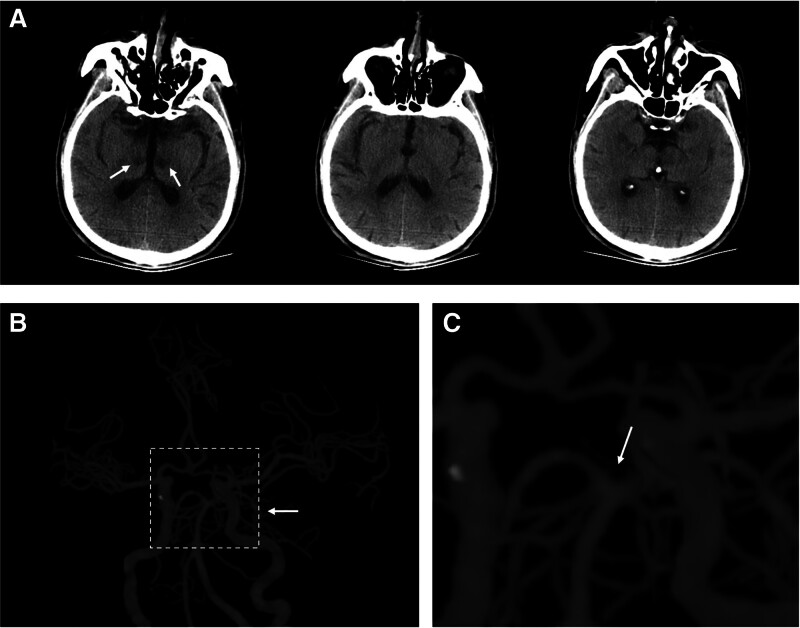
Brain imaging in the emergency room. (A) Coronal computed tomography showing bilateral hypodense lesions in thalamus (white arrows). (B) Computed tomographic angiography showing posterior cerebral artery variant, an arcade artery connecting bilateral posterior cerebral arteries, note white arrow in magnification shown in (C), and the distal of left posterior cerebral artery not clearly visualized (white arrow in B).

A 69-year-old cleaning worker presented to our emergency room with sudden onset of drowsiness for 2 days. The patient had no prior medical history, history of smoking or drinking, and no family or genetic diseases. He did not complain of fever, headache, trauma, or toxic exposure. His body temperature, blood pressure, peripheral oxygen saturation, and heart rate were all normal during the emergency room stay. On physical examination, he was well-oriented after awakening, with no obvious abnormalities found in the pupils, muscle strength, sensations, or bilateral Babinski signs. An emergency CT scan revealed bilateral hypodense thalamic lesions (Fig. 1A). Preliminary intracranial CT-angiography showed patency of his basilar artery, and interestingly, it detected an arcade artery connecting both PCAs, although the terminal portion of the left PCA was not clearly visualized (Fig. 1B and C). Bilateral thalamic infarction due to PCA variant was the first suspected diagnosis, and he was admitted to the department of neurology.

No significant abnormalities were found in the complete blood count, glucose tolerance test, lipid profile, liver, kidney, and thyroid function tests, B-type natriuretic peptide levels, arterial blood gases, coagulation screen, and venereal disease tests. The patient was placed under continuous electrocardiogram monitoring. Symptomatic therapies including aspirin, statins, and a gastric mucosal protective agent were also prescribed for the patient.

**Figure 2.**
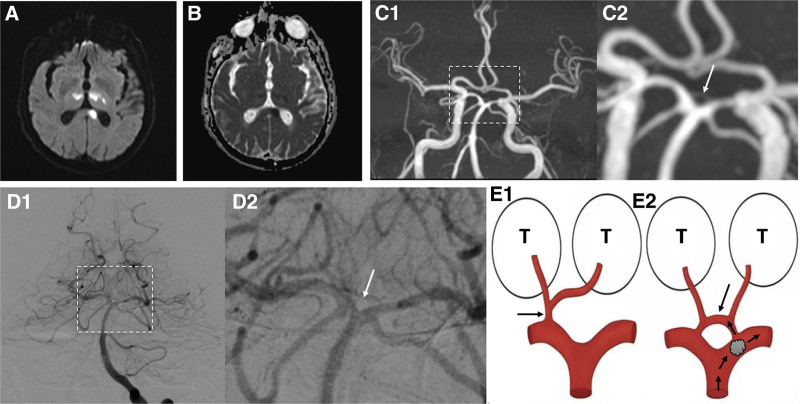
Brain magnetic resonance imaging (MRI) and digital subtraction angiography (DSA). (A and B) Axial diffusion-weighted MRI depicts cerebral infarction in left splenium of corpus callosum and bilateral thalamus with corresponding hypo-intensity in apparent diffusion coefficient sequence. (C and D) MR-angiography and DSA both detect the arcade artery (C1 and D1, magnification shown in C2 and D2 with white arrows). (E) Two uncommon variants (E1, artery of Percheron; E2, the arcade artery and potential stroke etiology in this patient; T = thalamus).

The brain MRI confirmed a cerebral infarction in the posterior circulation, which extended significantly beyond the territory of the paramedian thalamic artery (Fig. 2A and B). An embolic shower was the primary suspicion, given the accumulating infarcts across multiple vascular territories. MRA also revealed poor visualization of the left PCA, along with the arcade vessel (Fig. 2C). Additional DSA confirmed that imaging of the vascular bed in the posterior circulation using contrast enhancement was superior to MRA (Fig. 2D), indicating that a suspicious embolus was likely present at the junction between the arcade artery and the left PCA, which had further disintegrated and migrated (Fig. 2E).

**Figure 3.**
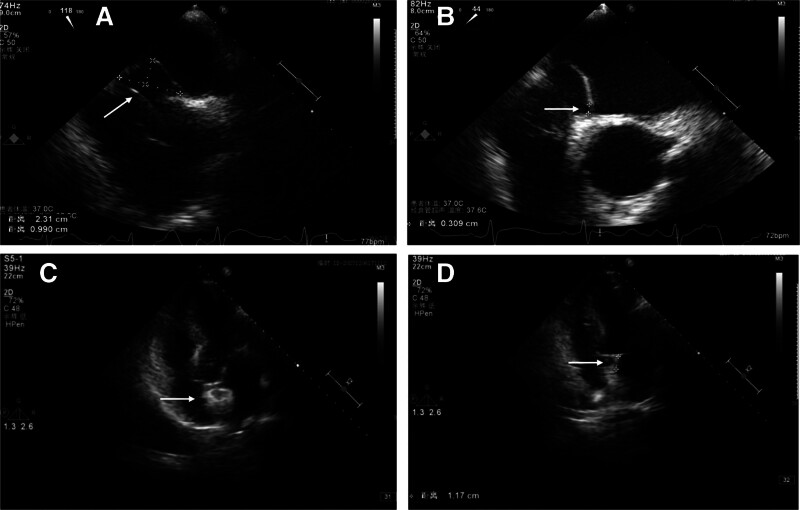
Transesophageal echocardiography (TEE) and ultrasound-guided foramen ovale interventional closure surgery. (A) TEE showing atrial septal bulge (23.1 mm × 9.9 mm, white arrow). (B) Patent foramen ovale (3.1 mm, white arrow). (C) Amplatzer occluder device (white arrow). (D) The distance between the occluder and the mitral valve root is 11.7 mm (white arrow).

The patient’s ambulatory electrocardiogram monitoring and 24-hour Holter electrocardiogram revealed a sinus rhythm. Within 24 hours, 14 atrial premature contractions and 14 ventricular premature contractions were noted. Transthoracic echocardiography showed mild regurgitation of the mitral, tricuspid, and aortic valves. Transesophageal echocardiography (Fig. 3A and B) and right ventriculography revealed a PFO measuring 3.1 mm, an atrial septal bulge, and an abnormally long Eurasian valve in the right atrium, which met the high-risk features for PFO. After injecting oscillating sterilized saline through the left elbow vein, bubbles were observed in the right atrium and right ventricle (Grade 3, >30 bubbles). Deep venous ultrasonography of the lower limbs revealed no signs of thrombosis. The tests for viruses, thrombophilia, anticardiolipin, lupus anticoagulants, protein C, protein S, and Factor V Leiden were all negative.

**Figure 4.**
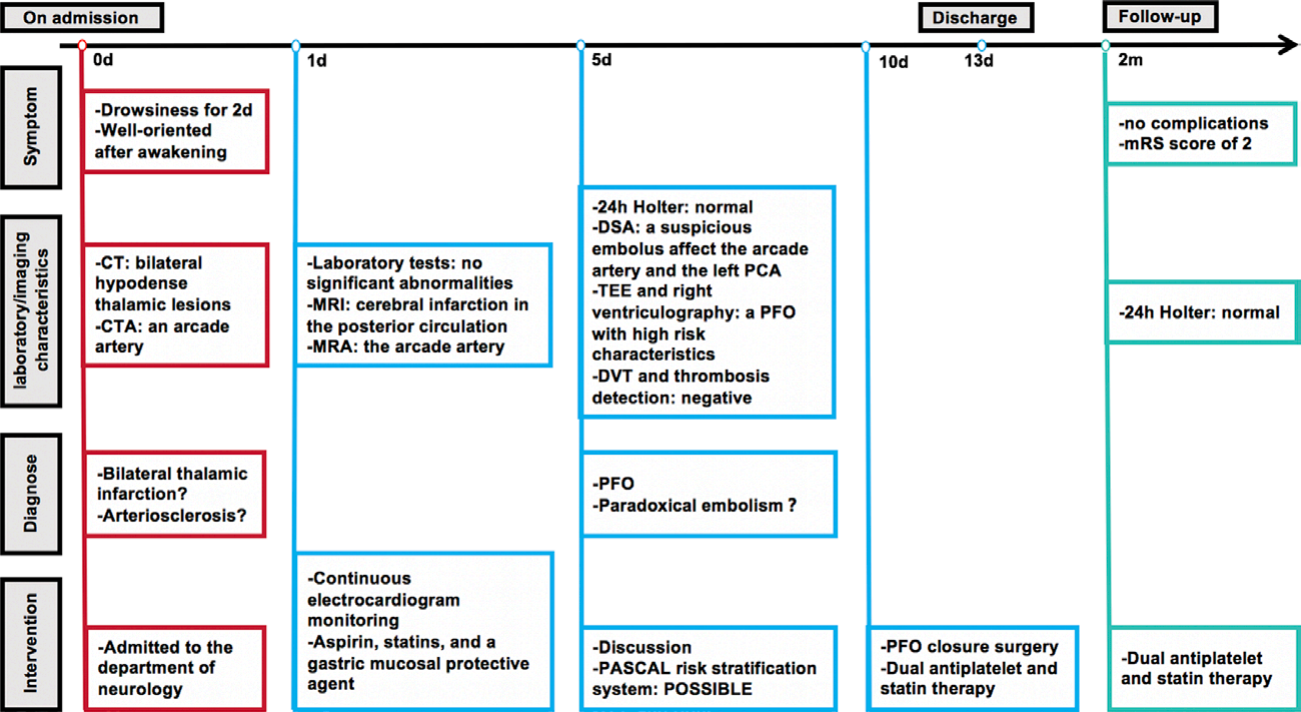
Timeline of the patient’s diagnosis and treatment process.

The patient was thoroughly discussed regarding the possibility of stroke recurrence following his preoperative evaluation using the prospectively validated PFO-associated stroke causal likelihood (PASCAL) risk stratification system.^[8]^ He had a Risk of Paradoxical Embolism (RoPE) score^[9]^ of 5. When combined with the PASCAL risk stratification system, the patient’s score was categorized as “POSSIBLE.” Theoretically, the risk of recurrent ischemic stroke could be reduced by approximately 62% following closure. This presented a diagnostic and therapeutic dilemma, and ultimately, he was sent for interventional PFO closure surgery (Fig. 3C and D), where he continued to receive dual antiplatelet and statin therapy. After 2 months of follow-up, he had no surgical or other complications, and the prognosis was favorable with a modified Rankin Scale score of 2. The timeline of the patient is presented in Figure 4.

## 3. Discussion

### 3.1. Bilateral thalamic infarction: why?

Posterior circulation ischemic strokes that involve the thalamus can present with a wide spectrum of unusual and variable clinical manifestations.^[10]^ Therefore, it is critical to have a thorough understanding of its anatomy and the possibility of corresponding arterial and vascular variants for diagnosis and treatment, particularly when attempting to explore the pathogenesis. This knowledge is essential for the development of secondary prevention strategies for patients.

The thalamus is a complex anatomical structure that serves as a subcortical center and sensory conduction relay station. Its functional activities influence the limbic system, motor system, ascending reticular system, and cerebral cortex. The variability in the clinical presentations of thalamic ischemic stroke, coupled with the multiple variations in blood supply, further complicates the appropriate identification of the condition during the acute phase.^[11]^

The thalamus is generally supplied by 4 groups of arteries: the thalamic tubercular artery, the paramedian thalamic artery, the inferior external thalamic artery, and the posterior choroidal artery.^[12]^ The paramedian thalamic artery supplies blood to the median region of the thalamus and has 4 common variants. In type I, the artery originates from the proximal end of the bilateral PCAs and supplies the ventromedial region of the thalamus on each side. In type IIa, the artery originates from the proximal PCA on one side and is responsible for the ventromedial thalamic supply to both sides. In type IIb, the artery originates from the P1 segment of a unilateral PCA and supplies both the ventromedial thalamus and the upper midbrain after branching, also known as the artery of Percheron (Fig. 2E). Type III arises from an arcade artery joining both PCAs (Fig. 2E).^[13]^ It is important to note that bilateral thalamic infarction is commonly detected in cases of unilateral or bilateral hypoplasia of the P1 segment.^[14]^ In this patient, the CT imaging mimicked as an artery of Percheron infarction.^[15]^ However, it was noteworthy that he had the type III variant, and the infarction focus clearly extended beyond the territory of the paramedian thalamic artery, implying involvement of the left PCA as well.

Timely MRI examination can help analyze the extent of infarction, the involvement of vascular territories, and differentiate it from basilar artery occlusion, which commonly involves the superior cerebellar arteries, the PCAs, and the pontine arteries. A diagnostic DSA can provide hints for categorization and etiology of a stroke. It was determined that the patient’s paramedian thalamic artery had a type III variant, connecting to both bilateral PCAs, and there was stenosis near the junction between the left portion of the arcuate vessel and the origin of the left PCA. Combined with the imaging findings, it strongly suggested a potential thromboembolic process. An easily disintegrative embolus ascended through a PFO, further migrated, and blocked the branches from the left portion of the arcuate artery and the left PCA (Fig. 2E).

### 3.2. Paradoxical embolism in the elderly: how?

Paradoxical embolism is an uncommon cause of ischemic stroke, in which emboli originate from the systemic venous circulation and enter the systemic arterial circulation through anatomic defects (e.g., PFO, atrial and ventricular septal defects) or extracardiac communications (e.g., pulmonary arteriovenous malformations).^[16]^ The role of PFO closure in individuals who have experienced a cryptogenic stroke at the age of 60 or older remains unclear due to the absence of randomized controlled trials that specifically address this age group. This knowledge gap is significant since moderate to severe PFO can be detected in up to 35% of cryptogenic stroke patients who are 65 years of age or older.^[17]^ Competing stroke mechanisms, such as atrial fibrillation, carotid artery disease, and valvular heart disease, are more prevalent in patients aged 60 and older, complicating the decision-making process. Furthermore, this age group has a higher prevalence of postoperative complications following PFO closure.^[18]^ Observational studies have demonstrated that well-selected older patients might also derive benefits from PFO closure, suggesting a potential beneficial role for PFO closure in the elderly population.^[6]^ However, because of the lack of robust data from clinical trials, current clinical guidelines and position statements do not uniformly recommend PFO closure for older patients who have experienced a cryptogenic ischemic stroke and have a PFO.

This case additionally demonstrates that older patients should be screened for PFO and tested for thrombophilia, especially in the absence of other stroke risk factors.^[19]^ Arterial ischemic stroke associated with PFO is a significant cause of disability and death in the elderly, and the treatment is a difficult clinical issue for neurologists, internists, or cardiologists. The RoPE score is considered to be a method of assessing a patient-specific “PFO attribution score,” the higher the score, the greater of probability that the stroke is associated with PFO.^[20]^ The new PASCAL risk stratification system^[8]^ categorizes patients into 3 subgroups based on 2 criteria: high-risk PFO features, which are defined as the presence of large shunts (>20–30 bubbles), atrial septal aneurysm (midline shift or complete shift), or both, and RoPE scores, which are assigned on a scale ranging from 0 to 6 or from 7 to 10. Patients with both high-risk PFO and a RoPE score of 7 or higher are classified as “PROBABLE.” Those “PROBABLE” patients experience a 90% reduction in the risk of recurrent ischemic stroke following closure. Patients are classified as “POSSIBLE” if they meet either of the following conditions: they have high-risk PFO but a RoPE score <7, or they have a low-risk PFO but a RoPE score of 7 or higher. “POSSIBLE” patients have a 62% lower risk of recurrent ischemic stroke after closure. Finally, if a patient has a low-risk PFO and a RoPE score of <7, they are classified as “UNLIKELY.” There was no significant difference between closure and medical therapy in patients classified as “UNLIKELY.” The patient received a score of “POSSIBLE” and interventional closure surgery was performed following multidisciplinary discussion based on his clinical and imaging findings. The patient is persistently given dual antiplatelet and statin therapy, and is still under our clinical follow-up.

## 4. Conclusion

Posterior circulation infarction, especially when accompanied by thalamic infarction, is characterized by elusive clinical manifestations and poor prognosis. Early identification and etiological analysis are essential although the causative mechanism of a stroke is always a probabilistic rather than definitive diagnosis. Moreover, the anatomical variation of patients may help attribute multiple and bilateral lesions to a single embolic source, thus facilitating the layered treatment strategy for secondary stroke prevention. This case surfaces the diagnostic and therapeutic dilemma of paradoxical embolism in the elderly population, however well-selected older patients might also derive benefits from PFO closure.

## Author contributions

**Conceptualization:** Langping Ling.

**Data curation:** Lingjia Xu, Yang Zhou.

**Investigation:** Yang Zhou.

**Supervision:** Langping Ling, Lingjia Xu.

**Validation:** Langping Ling.

**Writing – original draft:** Langping Ling, Lingjia Xu, Yang Zhou.

**Writing – review & editing:** Langping Ling, Lingjia Xu, Yang Zhou.

## References

[1] Ide S Kakeda S Korogi Y. [Anatomy of the Thalamus]. Brain Nerve. 2015;67:1459–69. 26618760 10.11477/mf.1416200323

[2] Schmahmann JD. Vascular syndromes of the thalamus. Stroke. 2003;34:2264–78. 12933968 10.1161/01.STR.0000087786.38997.9E

[3] Amici S. Thalamic infarcts and hemorrhages. Front Neurol Neurosci. 2012;30:132–6. 22377880 10.1159/000333611

[4] Lin G Zhang X Hu B. Paramedian thalamic ischemic infarction: a retrospective clinical observation. Eur Neurol. 2017;77:197–200. 28190011 10.1159/000458705

[5] Aggeli C Verveniotis A Andrikopoulou E Vavuranakis E Toutouzas K Tousoulis D. Echocardiographic features of PFOs and paradoxical embolism: a complicated puzzle. Int J Cardiovasc Imaging. 2018;34:1849–61. 29956022 10.1007/s10554-018-1406-1

[6] Wintzer-Wehekind J Alperi A Houde C. Transcatheter closure of patent foramen ovale in patients older than 60 years of age with cryptogenic embolism. Rev Esp Cardiol (Engl Ed). 2020;73:219–24. 31585849 10.1016/j.rec.2019.07.003

[7] Scacciatella P Jorfida M Biava LM. Insertable cardiac monitor detection of silent atrial fibrillation in candidates for percutaneous patent foramen ovale closure. J Cardiovasc Med (Hagerstown). 2019;20:290–6. 30921267 10.2459/JCM.0000000000000790

[8] Sposato LA Albin CSW Elkind MSV Kamel H Saver JL. Patent foramen ovale management for secondary stroke prevention: state-of-the-art appraisal of current evidence. Stroke. 2024;55:236–47. 38134261 10.1161/STROKEAHA.123.040546

[9] Kent DM Ruthazer R Weimar C. An index to identify stroke-related vs incidental patent foramen ovale in cryptogenic stroke. Neurology. 2013;81:619–25. 23864310 10.1212/WNL.0b013e3182a08d59 PMC3775694

[10] Goerlitz J Wenz H Al-Zghloul M Kerl HU Groden C Förster A. Anatomical variations in the posterior circle of willis and vascular pathologies in isolated unilateral thalamic infarction. J Neuroimaging. 2015;25:983–8. 25786673 10.1111/jon.12235

[11] Li S Kumar Y Gupta N. Clinical and neuroimaging findings in thalamic territory infarctions: a review. J Neuroimaging. 2018;28:343–9. 29460331 10.1111/jon.12503

[12] Bordes S Werner C Mathkour M. Arterial supply of the thalamus: a comprehensive review. World Neurosurg. 2020;137:310–8. 32036065 10.1016/j.wneu.2020.01.237

[13] Percheron G. The anatomy of the arterial supply of the human thalamus and its use for the interpretation of the thalamic vascular pathology. Z Neurol. 1973;205:1–13. 4126735 10.1007/BF00315956

[14] Jiménez Caballero PE. Bilateral paramedian thalamic artery infarcts: report of 10 cases. J Stroke Cerebrovasc Dis. 2010;19:283–9. 20610185 10.1016/j.jstrokecerebrovasdis.2009.07.003

[15] Lazzaro NA Wright B Castillo M. Artery of percheron infarction: imaging patterns and clinical spectrum. AJNR Am J Neuroradiol. 2010;31:1283–9. 20299438 10.3174/ajnr.A2044 PMC7965474

[16] Windecker S Stortecky S Meier B. Paradoxical embolism. J Am Coll Cardiol. 2014;64:403–15. 25060377 10.1016/j.jacc.2014.04.063

[17] Mazzucco S Li L Rothwell PM. Prognosis of cryptogenic stroke with patent foramen ovale at older ages and implications for trials: a population-based study and systematic review. JAMA Neurol. 2020;77:1279–87. 32628255 10.1001/jamaneurol.2020.1948 PMC7550974

[18] Healey JS Alings M Ha A. Subclinical atrial fibrillation in older patients. Circulation. 2017;136:1276–83. 28778946 10.1161/CIRCULATIONAHA.117.028845

[19] Mac Grory B Ohman EM Feng W. Advances in the management of cardioembolic stroke associated with patent foramen ovale. Bmj. 2022;376:e063161. 35140114 10.1136/bmj-2020-063161

[20] Strambo D Sirimarco G Nannoni S. Embolic stroke of undetermined source and patent foramen ovale: risk of paradoxical embolism score validation and atrial fibrillation prediction. Stroke. 2021;52:1643–52. 33784832 10.1161/STROKEAHA.120.032453

